# Exploring the Relationship Between Learning Goal Orientation and Knowledge-Sharing Among Information Communication Technology Consultants: The Role of Incentive Schemes

**DOI:** 10.3389/fpsyg.2022.798668

**Published:** 2022-02-09

**Authors:** Linpei Song, Zhuang Ma, Jun Huang

**Affiliations:** ^1^School of Business Administration, Gachon University, Seongnam-si, South Korea; ^2^International Business School, Chongqing Technology and Business University, Chongqing, China; ^3^School of Economics and Management, Southwest University, Chongqing, China

**Keywords:** knowledge-sharing, learning goal orientation, ICT consultants, goal orientation, motivation theory, incentive schemes

## Abstract

Knowledge sharing (KS) is critical for consulting companies to develop sustainable competitive advantages. While the importance of KS in the information communication technology (ICT) sector has been proved, the assumed linear relationships in KS mechanisms are confronted with KS dilemmas: consultants’ intention to maximize personal gains from KS resulting in restrained KS efforts, for fear of losing value after sharing knowledge with colleagues. Drawing on motivation theory and goal orientation perspective, this study examines the roles of learning goal orientation (LGO) and incentive schemes in KS among ICT consultants. The multiple regression analyses of 389 consultants’ responses from 14 Chinese and 8 Korean ICT consulting companies demonstrated an inverted U-shape relationship between LGO and knowledge sharing; incentive schemes moderate this relationship. The findings shed light on the knowledge-sharing dilemma, with theoretical implications to research regarding goal-orientation, knowledge sharing, and managerial practices about the motivation and incentives of ICT consultants.

## Introduction

Knowledge can facilitate decision making improve organizational effectiveness and innovation, and thus has become a critical success factor and source of a firm’s competitive advantage (Matzler and Mueller, 2011; Chen et al., 2018). Knowledge can be developed from experienced employees, shared during their interactions with colleagues, and transferred from one department to another (Matzler and Mueller, 2011). Managers have endeavored to convert employees’ knowledge into knowledge that can be articulated, codified, and shared within organizations (Tsai and Cheng, 2012). Knowledge sharing has received increasing attention from information systems researchers (Tsai and Cheng, 2012), possibly because ICT firms can adapt available technologies to facilitate knowledge sharing among employees (Tsai and Cheng, 2012; Eisenbardt, 2021). Knowledge sharing in this study refers to the activities (e.g., workshops and mentoring) where ICT consultants share their knowledge with colleagues within the same firm (Cui, 2017).

Effective knowledge sharing within ICT firms can be difficult and complex to achieve (Alavi and Leidner, 2001; Pereira and Mohiya, 2021; Venkatesh et al., 2021). Given such difficulties, it is worth investigating the underlying mechanisms that affect knowledge sharing among employees. So far, several studies have investigated the individual-level factors (e.g., proactiveness and motivation) that lead to knowledge sharing, probably because of the knowledge sharing dilemma (i.e., a social situation where employees fear that sharing acquired knowledge to colleagues may eventually harm their benefits (e.g., status, promotional opportunities), thus deciding to hide knowledge (Cabrera and Cabrera, 2002; Pereira and Mohiya, 2021). However, knowledge sharing involves a reciprocal process where employees share some knowledge to reciprocate with colleagues (Venkatesh et al., 2021). In this case, a goal, i.e., what employees seek to achieve, can motivate employees to proactively take learning activities to acquire new knowledge and at the same time share knowledge (Matzler and Mueller, 2011). As a motivational variable, learning goal orientation (LGO) has been adopted to explain employees’ learning and adaptive behavior for various tasks (Shariq et al., 2019; Lim and Shin, 2020), such as knowledge sharing. While several studies (Zacher and Jimmieson, 2013; Islam et al., 2020) suggest that learning goal-oriented employees are more self-driven toward sharing newly acquired knowledge, the social dilemmas involved in sharing knowledge (Cabrera and Cabrera, 2002) suggest that a beneficiary of the knowledge shared by other consultants may consider such knowledge as a public good and thus unwilling to reciprocate. Accordingly, LGO might not always lead to knowledge sharing among ICT consultants.

This study intends to provide an improved understanding of the antecedents and interactive mechanisms that affect knowledge sharing. It integrates the impacts of both the motivational (i.e., ICT consultants with LGOs) and corporate (i.e., incentive schemes) (Wolfe and Loraas, 2008) level factors to on employees’ knowledge sharing activities. Previous studies suggest that employees who receive rewards are more likely to share knowledge (Baron James and Kreps David, 1999; Zárraga and Bonache, 2003; Saether, 2020). However, it is unclear how employees weigh such incentives against the costs (e.g., losing customers and promotional opportunities) and misgivings (e.g., the recipients may not reciprocate) of sharing specific knowledge to colleagues. Drawing on the insights about knowledge sharing in ICT firms, employee’s LGO, and incentive schemes in the above literature, this study adopts the motivation-knowledge-sharing mechanism, with incentive schemes as a potential moderator, to answer the following research questions:

RQ1: How does an ICT consult’ LGO affect his or her knowledge sharing activities?

RQ2: How do an ICT consulting firm’s incentive schemes affect the above relationship?

The rest of this paper is structured as follows: The next section presents the literature review and hypothesis development process, with section “Materials and Methods” providing the research approach, methods, and measures of variables. Section “Results” presents the results of the study, followed by Section “Conclusion,” which discusses the theoretical implications, managerial implications, limitations, and future search. The last section concludes this study.

## Theoretical Background and Hypotheses

### Knowledge Management in Information Communications Technology Consulting Firms

ICT consulting firms providing knowledge-intensive business services (KIBS), i.e., firms providing services [e.g., information and communications technology (ICT), consulting, and R&D], often require technological and specialized knowledge (Dobrai and Farkas, 2009). As their customers’ businesses grow more complex and diverse, KIBSs may face the challenges to develop specialized and in-depth knowledge. The knowledge-intensive nature of KIBSs suggests that these firms rely on the knowledge and expertise (e.g., analytical and social skills) of their experts (e.g., agents and consultants) to develop and deliver services (Frenkel et al., 1995; Hassan, 2017).

ICT consulting firms often rely on their consultants to develop ICT services that meet customers’ specific needs, optimize the functionality of customers ICT investment, impart best practices to customers’ employees, and help customers achieve digital strategic transformation. In order to ensure service performance, ICT consulting firms need to implement knowledge management initiatives to stimulate knowledge learning, storing, and sharing among their consultants (van Zyl et al., 2019).

In this case, consultants are expected to acquire and share the information, ideas, suggestions, and expertise relevant to the firm’s businesses within their employers (Hassan, 2017). While explicit knowledge can be easily developed, articulated, codified, and shared among consultants (Tsai and Cheng, 2012), implicit and tacit knowledge is often learned and accumulated through long-term experience, thus hard to codify and share (Choo, 2000). In addition, the learning and sharing of implicit and tacit knowledge require continuous efforts and may involve costs for employees and the firm (Grant, 1996). As such, how ICT consulting firms motivate consultants to continuously upgrade their knowledge and share the various forms of knowledge with colleagues is worth investigating.

### Learning Goal Orientation as a Motivational Variable

To achieve sustainable performance, firms need employee development programs to upgrade the skills and knowledge of their employees (Mumford, 2000; Rasool et al., 2019). However, the effectiveness of employee development programs could vary according to employee characteristics such as motivations. Goals are widely recognized as being central to the understanding of motivations, with different research disciplines emphasizing different levels and types of goals and their consequences (Brett and VandeWalle, 1999). A goal can be understood as an aim that one is committed to and serves as a predictor for future behavior (Wigfield and Cambria, 2010). A clear goal allows an employee to understand his/her work orientation, motives, values, and abilities related to work (Messarra et al., 2009), therefore explaining his/her knowledge acquisition in employee development programs. Rooted in educational psychology, goal orientations can drive individuals to learn new knowledge.

Goal orientations include LGO and performance goal orientation (PGO) (Dweck and Leggett, 1988). Individuals with LGOs tend to involve themselves in positive efforts and persistence in a challenging situation, and their satisfaction comes from the mastery or completion of a task (DeShon and Gillespie, 2005). Learning-goal oriented employees tend to improve their abilities and build new skills to improve performance (Shariq et al., 2019). For instance, learning-goal oriented professionals such as ICT consultants are self-driven to develop and use their technical expertise to develop new services, solve problems and address challenges at work (Messarra et al., 2009). Successful experience in addressing difficulties and acquiring new knowledge can further raise employees’ ambition to exert more effort on learning (Zhou et al., 2021). In contrast, individuals with PGOs tend to compare with others in terms of their abilities, such as undertaking tasks better than others (Urdan, 1997). Individuals with performance-oriented goals often seek favorable feedbacks on their abilities and avoid negative comments about their abilities (Wigfield and Cambria, 2010). Under PGO, employees are driven by the comparison with colleagues in terms of performance and the perceived expectation from supervisors; as such, they are less likely to challenge themselves with challenging tasks and learning activities (Shamim et al., 2017). Due to dependence on supervisors, an ICT consultant with a performance-goal orientation may lack the autonomous and entrepreneurial spirit to independently acquire knowledge and develop new services or help other colleagues by sharing their expertise (Messarra et al., 2009). Moreover, LGO is more related to focused and adaptive behaviors, while PGO is more related to ego and defensive behaviors (Dweck and Leggett, 1988). Several scholars have further suggested that individuals in a learning goal-orientated context are more likely to engage in proactive learning behaviors; in contrast, in a performance-oriented context have no consistent relationship with learning behaviors (Roeser et al., 1996; Bunderson and Sutcliffe, 2003). In other words, learning goals rather than performance goals tend to predict adaptive behaviors, attitudes, and outcomes (DeShon and Gillespie, 2005). As this study aims to investigate how goal orientation drives employees into knowledge sharing, our focus is on LGO.

### Learning Goal Orientation and Knowledge Sharing

The precedents of knowledge sharing have been investigated extensively. Several studies have focused on the organizational level factors, such as corporate culture, human resource management practices, social capital, relationships, and organizational pessimism (Mueller, 2014; Rasool et al., 2019; Stojanović-Aleksić et al., 2019; Asgari et al., 2020). In contrast, another stream of literature stresses individual-centric antecedents such as individual motivation. In terms of knowledge sharing, employees could be more motivated to share knowledge once they perceive rewarding outcomes, such as opportunities for professional growth and creativity (Chumg et al., 2015; Nguyen et al., 2019). Some studies (Suppiah and Sandhu, 2011; Kwahk and Park, 2016; Zhou et al., 2021) suggest that perceived self-efficacy and perceived self-enjoyment could enable employees to develop a sense of improving knowledge (i.e., achievement) and help colleagues as altruistic behavior (mental well-being). Likewise, some employees would share knowledge as a form of reciprocity, i.e., sharing knowledge with colleagues and expecting to learn from these colleagues in the future (Schulz, 2001; Schmid and Schurig, 2003). The reciprocal knowledge exchange relationship encourages knowledge sharing behavior, and as a result, individuals may be more willing to share their valuable knowledge (Zimmermann and Ravishankar, 2014). As a motivational factor, LGO could also promote knowledge sharing among employees. Compared to colleagues with low LGO, ICT consultants with a high degree of learning-goal orientation could be more motivated to constantly develop and upgrade their knowledge to benefit themselves and exchange with colleagues (Schulz, 2001; Schmid and Schurig, 2003). However, after a certain point, a high degree of LGO may no longer be helpful to knowledge sharing. For one thing, knowledge sharing involves a complex process of assimilation, compilation, enhancement, and transfer (Obrenovic et al., 2020). While learning-goal oriented ICT consultants may have the motivation to acquire new knowledge, they may find it challenging to share it with colleagues, especially when the acquired knowledge (e.g., tacit knowledge) grows in depth and difficulty. For another, knowledge sharing among employees depends largely on employees’ willingness (Obrenovic et al., 2020). As knowledge upgrades from fundamental to critical, employees, especially those from knowledge-intensive firms, may tend to keep the new knowledge to themselves as a kind of property (Amble, 2006). In particular, ICT consultants may become demotivated to share knowledge when they realize that sharing specific knowledge may affect their sense of power and competitive advantage at work. When an ICT consultant proactively shares knowledge or information with colleagues, he or she expects a reciprocal contribution from others, so he/she could benefit from such an exchange. However, the social dilemma in knowledge sharing suggests that the shared knowledge is accessible to all the consultants (including the knowledge contributors), the non-contributors could be tempted to adopt the new knowledge at work without sharing their own knowledge (Sweeney, 1973; Cabrera and Cabrera, 2002).

Although the relationship between LGO and knowledge sharing is positive at lower levels of LGO, it may become weaker and eventually disappear at higher levels of the construct. Beyond this threshold, higher LGO may no longer be related to knowledge sharing. Therefore, the following can be proposed:

Hypothesis 1: A curvilinear relationship exists between LGO and knowledge sharing, such that the relationship is initially positive but becomes less positive as LGO increases; the relationship disappears as LGO increases further.

### The Moderating Effect of Incentive Schemes

Despite the hypothesized relationship between LGO and knowledge sharing, the pattern of this relationship is contingent on different contexts. Rasool et al. (2021) examine how a toxic workplace environment presents the atmosphere that negatively affects employee wellbeing and employee engagement, and suggest that firms’ support could improve employee engagement. In particular, firms’ support could encourage employee contribution, and elevate employees’ cognitive and emotional appraisals of their employers (Wang et al., 2020; Rasool et al., 2021). Drawing on the motivation theory (Nguyen et al., 2019), we expect a curvilinear relationship between LGO and knowledge sharing under the condition of incentive schemes. Well-designed incentive schemes could shape employees’ behavior by articulating the kinds of behavior appreciated and regarded as valuable by the organization (Cabrera and Bonache, 1999; Rasool et al., 2019). Incentive schemes could include monetary incentives (e.g., pay and remuneration) and non-monetary incentives (e.g., medical aid, learning opportunities, and desirable work environment) (Moore and Bussin, 2012). Both monetary and non-monetary incentives could motivate employees by addressing their various physiological and psychological needs (Wolfe and Loraas, 2008). Monetary and incentives could meet employees’ physiological (e.g., improved living standards) and psychological needs (improved status as compared with colleagues). Therefore, in ICT consulting firms where incentive schemes for knowledge sharing are available, learning-goal oriented employees are more likely to improve and share new knowledge within the organization. Accordingly, the following can be proposed: Hypothesis 2: Incentive schemes moderate the relationship between LGO and knowledge sharing.

### The Interactive Effect Between Learning Goal Orientation and Incentive Schemes on Knowledge Sharing

This study investigates the combined effects of LGO and incentive schemes on knowledge sharing. As is hypothesized above, the relationship between LGO and knowledge sharing is moderated by incentive schemes. However, Antoni et al. (2017) warn that incentive schemes may not always motivate employees and may sometimes waste money and generate little motivation for employees. Indeed, incentive schemes may motivate ICT consultants to learn new knowledge related to their work. However, before sharing knowledge with their colleagues, these consultants may compare the expected incentives with the cost of sharing the acquired knowledge with colleagues. When an ICT consultant has acquired explicit knowledge (e.g., routines and templates), they are more likely to share it through project documents, emails, and meetings (Hassan, 2017). The consultant is willing to share such explicit knowledge as it can bring back monetary incentives (e.g., prize money in idea generation meeting) or non-monetary incentives (e.g., praises from superiors and appreciations from new colleagues) while not compromising his or her core competitive advantage. However, when the acquired knowledge upgrades from explicit to implicit and then to tacit knowledge, the ICT consultant would consider it more costly to share it. Such knowledge involves valuable perceptions and insights developed from long-term experience and is rarely expressed openly (Smith, 2001). When an ICT consultant realizes that he/she possess new knowledge that other colleagues (and even the superiors) do not have, he/she may worry that sharing such knowledge could lead to higher costs of knowledge sharing than the increased incentives. This is particularly true when a consultant finds that his or her knowledge sharing attitudes are not comparable to the benefits from the firm’s incentive schemes. Therefore, under conditions of low incentive schemes, employees with a low degree of LGO are more willing to share knowledge for their personal gains (e.g., promotion and pay raise); in contrast, under conditions of the high incentive schemes, employees with a high degree of LGO are unwilling to share knowledge, for fear of a higher cost. Accordingly, the following can be proposed:

Hypothesis 3: The interaction effect between the quadratic term of LGO and incentive schemes has a significant impact on knowledge sharing, such that the level of LGO at which its relationship with knowledge sharing disappears (i.e., the inflection point) is determined by incentive schemes such that the inflection point for more incentive schemes is likely to occur at lower levels of LGO than the inflection point for less incentive schemes.

**FIGURE 1:**
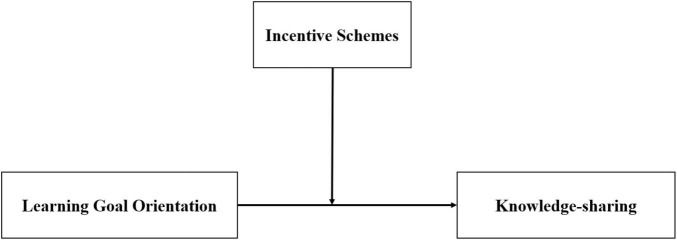
Theoretical framework.

The above hypotheses lead to a theoretical framework (see Figure 1), which we further tested in this study.

## Materials and Methods

### Research Approach

This empirical study follows a quantitative strategy to analyze the impacts of LGO on knowledge sharing in ICT consulting firms and the moderating role of incentive schemes in this relationship. It follows the deductive and cross-sectional approach, which supports the development of hypotheses based on established theories and designing the research strategy to test the hypotheses (Wilson, 2014). Cross-sectional research uses cross-sectional data, which is commonly adopted in social sciences (Li et al., 2018; van Zyl et al., 2019). We adopted the questionnaire survey method, which allows us to effectively access a large sample of the target population (Rasool et al., 2021; Zhou et al., 2021).

### Questionnaire Development

Drawing on the literature review, we developed a theoretical framework related to LGO, knowledge sharing, and incentive schemes. In this framework, LGO influences ICT consultants’ knowledge sharing, with this relationship moderated by incentive schemes. During the questionnaire design, we prepared the Chinese version and Korean version for respondents from China and South Korea. We then conducted the pilot test to the translated versions to ensure reliability and validity. The pilot test involved inviting two managers, two Ph.D. students, and one professor from China, and two managers and two Ph.D. students from South Korea. Those participants suggested some revisions and corrections to avoid misunderstanding, and they did not participate in the final survey. Finally, we distributed the questionnaire to the human resource (HR) departments of ICT consulting firms from China and South Korea.

#### Measures

All items were measured on 5-point-Likert-scales (1: strongly disagree; 5: strongly agree).

#### Learning Goal Orientation

The scale for the independent variable, i.e., LGO, was adapted from Matzler and Mueller (2011). The three items in this scale were revised and adjusted to meet the research scope of this study. The items include, “Accomplishing a tough project is very satisfying,” “An important part of being a good employee is continually improving our skills” and “I put in a great deal of effort sometimes in order to learn something new related to my job.” The Cronbach alpha of this study was 0.91.

#### Knowledge Sharing

The scale for the dependent variable, i.e., Knowledge Sharing (KS), was adapted from Matzler and Mueller (2011). The five items in this scale were used to measure knowledge sharing among employees. These items include “I often share general topics (e.g., goals and budgets) with colleagues at work,” “I often share project specific requirements (e.g., project data, deadlines, and project rations) with colleagues at work,” “[I often share methods and techniques (e.g., new techniques, methods, and failures) with colleagues at work],” “I often share important knowledge (customer insights and new opportunities) with colleagues at work,” and “I often share project results (e.g., preliminary results, unexpected outcomes, and recommendations) with colleagues at work.” The Cronbach alpha of this study was 0.93.

#### Incentive Schemes

As we focus incentive schemes (IS) related to knowledge sharing, we measured this moderating variable by adapting the reward systems linked to the knowledge sharing scale in Zárraga and Bonache (2003). The four items were: “A variable part of my pay depends on my colleagues” assessment of the degree to “which I cooperate with them,” “My bonus partly depends on the results that my team/firm achieves,” “A significant part of my salary depends on the overall performance of my colleagues,” and “My company rewards and compensates those employees who help their colleagues to improve and develop.” The Cronbach alpha of this study was 0.88.

#### Control Variables

In selecting variables to include as controls, we considered the variables that might provide alternative explanations for knowledge sharing behavior or employee job performance. We controlled for age, gender, tenure, education, occupation, and industry, which are widely accepted predictors of employee performance (Button et al., 1996; Ng and Feldman, 2010).

### Sample and Data Collection

The sample included 389 employees from Chinese and Korean ICT consulting firms. The human resource (HR) departments of 8 ICT consulting firms from Korea and 14 ICT from China were approached, each receiving an invitation letter describing the purpose of this study. Eventually, 22 firms agreed to participate. Data were collected between the 1st of September 2020 and the 20th of February 2021. We first contacted consultants via work email to seek their agreement to participate. A total of 565 employees were sent an invitation by their HR departments to volunteer for the study, out of whom 532 offered to participate. The HR administrators distributed and collected the questionnaires to ensure confidentiality. Survey links were sent to the respondents’ email accounts to complete the survey. A total of 412 surveys were returned, giving a response rate of 77%.

### Demographics

Table 1 presents the demographic information of our samples. In terms of the 389 respondents’ gender, 55% were male and 45% female, generally suggesting a balance between each gender. Regarding age groups, 40% were aged between 25 and 30, 27% between 31 and 35, and 18% between 36 and 40, with 16% over 41 years old. Such distribution aligned with the larger proportion of young employees in ICT consulting firms. In terms of education, 23% had an Associate degree or below, 51% had a Bachelor’s degree, 23% had a Master’s degree, and 4% had a Doctorate degree. In terms of nationality, 52% were Chinese, and 48% were Korean.

**TABLE 1:** Demographics of respondents.

Characteristics	Category	Frequency	Percentage
Gender	Male	215	55.3%
	Female	174	44.7%
Age	25–30	156	40.1%
	31–35	104	26.7%
	36–40	68	17.5%
	41 above	61	15.7%
Edu	Associate degree or below	89	22.9%
	Bachelor degree	197	50.6%
	Master degree	88	22.6%
	Doctorate degree	15	3.9%
Nationality	China	204	52.4%
	South Korea	185	47.6%

## Results

We transferred the data into IBM SPSS Statistics 25 to examine outliers, missing values, normality, and multicollinearity. We designed the questionnaire in a way that the respondents had to answer all the questions in order to submit the questionnaire. This allowed us to avoid missing values. We then reported the descriptive analysis before the confirmatory factor analysis (CFA) to test the reliability and validity of each instrument. Finally, we conducted a multiple regression analysis to test the hypotheses.

### Sample Description

Descriptive analysis of the data distribution is present in Table 2. The descriptive measures include the mean and standard deviation. Each item is gauged through a 5-point Likert scale. The mean values of the items are between 3.53 and 3.86. The standard deviation values for all items fall in the range of 0.889–1.223.

**TABLE 2:** Descriptive analysis.

No	Codes	Items	N	Min.	Max.	Mean	SD
1	LGO1	Accomplishing a tough project is very satisfying.	389	1	5	3.53	0.978
2	LGO2	An important part of being a good employee is continually improving my skills.	389	1	5	3.57	1.025
3	LGO3	I put in a great deal of effort sometimes in order to learn something new related to my job.	389	1	5	3.61	1.016
4	KS1	I often share general topics (e.g., goals and budgets) with colleagues at work.	389	1	5	3.69	1.223
5	KS2	I often share project specific requirements (e.g., project data, deadlines, and project rations) with colleagues at work.	389	1	5	3.65	1.136
6	KS3	I often share methods and techniques (e.g., new techniques, methods, and failures) with colleagues at work.	389	1	5	3.85	0.901
7	KS4	I often share important knowledge (customer insights and new opportunities) with colleagues at work.	389	1	5	3.68	1.172
8	KS5	I often share project results (e.g., preliminary results, unexpected outcomes, and recommendations) with colleagues at work.	389	1	5	3.73	0.889
9	IS1	A variable part of my pay depends on my colleagues’ assessment of the degree to which I cooperate with them.	389	1	5	3.77	0.986
10	IS2	My bonus partly depends on the results that my team/firm achieves.	389	1	5	3.86	0.990
11	IS3	A significant part of my salary is due to the overall performance of my colleagues.	389	1	5	3.72	1.051
12	IS4	My company rewards and compensates those employees who help their colleagues to improve and develop.	389	1	5	3.77	1.091

*Total items, 12; Learning Goal Orientation (LGO), 3; Knowledge-sharing (KS), 5; Incentive Scheme (IS), 4.*

### Validity and Reliability

We conducted a CFA to examine the composite reliability, convergent validity, and discriminant validity of the instruments. Convergent validity was ensured with composite reliability (CR) above 0.80 and AVEs over 0.50 (Fornell and Larcker, 1981). Table 3 shows that all CRs are higher than the suggested 0.80 and all AVE values are higher than the suggested 0.50, indicating a good convergent validity of the measurement model. The square roots of factors’ AVEs were higher than their correlation coefficients with other factors that strongly support the discriminant validity (Fornell and Larcker, 1981) in Table 4.

**TABLE 3:** Results of validity and reliability tests.

Variables	Cronbach’s alpha	Std. factor loading	CR	AVE
LGO	0.91	0.87	0.91	0.77
		0.86		
		0.90		
KS	0.93	0.86	0.94	0.76
		0.80		
		0.88		
		0.93		
		0.87		
IS	0.88	0.79	0.89	0.66
		0.80		
		0.84		
		0.82		

*LGO, learning goal orientation; KS, knowledge sharing; IS, incentive scheme.*

**TABLE 4:** Results of correlation and discriminant validity analysis.

		Mean	SD	AVE	1	2	3	4	5	6	7
1	Gender	1.45	0.50	−	1.						
2	Age	2.09	1.09	−	–0.03	1.					
3	Nationality	1.48	0.50	−	0.09	–0.05	1.				
4	Edu	2.07	0.78	−	0.00	–0.05	0.07	1.			
5	LGO	3.57	0.93	0.77	0.01	–0.05	−0.11*	–0.03	0.88		
6	KS	3.72	0.95	0.76	–0.02	0.00	–0.07	–0.03	0.38**	0.87	
7	IS	3.78	0.89	0.66	–0.03	0.00	0.02	0.04	0.12*	0.12*	0.81

*p < 0.05; **p < 0.01.

We also adopted a comparison model to examine the discriminant validity in Table 5. The three-factor model fitted the data well, χ^2^/df = 1.346, Comparative fit index (CFI) = 0.995, Tucker–Lewis index (TLI) = 0.993, Root mean square error approximation (RMSEA) = 0.030, Standardized root mean square residual (SRMR) = 0.024, while the other factor models revealed a poor fit for the data. And the model fit was not significantly improved by adding the common-method bias factor; the common method variance is not a major issue (Williams et al., 1989). To summarize, the three-factor model demonstrated adequate reliability and validity.

**TABLE 5:** Results of discriminant validity.

Comparison model	χ ^2^	df	χ ^2^/df	CFI	TLI	RMSEA	SRMR
Common method bias model	68.455	50	1.369	0.995	0.993	0.031	0.026
Three-factor model	68.632	51	1.346	0.995	0.993	0.030	0.024
Two-factor model	748.081	53	14.115	0.795	0.744	0.184	0.117
One-factor model	1565.621	54	28.993	0.553	0.454	0.268	0.203

*Three-factor model (LGO, KS, IS); Two-factor model (LGO + KS, IS); One-factor model (LGO + KS + IS).*

### Correlation Analysis

The means, standard deviations, and intercorrelations of all variables used in this study are provided in Table 4. In the correlation matrix, LGO was significantly and positively correlated with knowledge sharing (*r* = 0.38, *p* < 0.05), partial support for our hypotheses. In addition, incentive scheme was significantly correlated with LGO (*r* = 0.12, *p* < 0.05), knowledge sharing (*r* = 0.12, *p* < 0.05).

### Hypothesis Testing

Table 6 shows the analysis results regarding the relationships between LGO and knowledge sharing. To address multicollinearity, we used standardized values of the independent variables (described above) in all the regression models (Aiken et al., 1991). As can be seen, the quadratic effect of LGO in Step 2 for the regression model predicting knowledge sharing was statistically significant (β = −0.19, *p* < 0.01), supporting Hypothesis 1. The signs of the quadratic effects were negative for LGO and knowledge sharing, indicating that the relationships resemble an inverted-U shape. This means that an increase in LGO will initially lead to knowledge sharing, but the relationships will become weaker and eventually disappear when LGO increases past a certain point. And in Step 3, it can be found that Hypothesis 2 regarding the moderating effect of the incentive scheme was also supported.

**TABLE 6:** Examining the relationships between LGO and KS as Moderated by IS.

Predictor	Knowledge sharing		Knowledge sharing		Knowledge sharing		Knowledge sharing	
	β	SE.	β	*SE*.	β	*SE*.	β	*SE*.

	Step 1		Step 2		Step 3		Step 4	
Gender	–0.01	0.10	–0.02	0.09	–0.01	0.09	–0.01	0.09
Age	0.00	0.04	0.02	0.04	0.02	0.04	0.02	0.04
Nationality	–0.07	0.10	–0.02	0.09	–0.01	0.09	–0.02	0.09
Edu	–0.03	0.06	–0.02	0.06	–0.03	0.06	–0.02	0.06
LGO			0.29***	0.05	0.28***	0.05	0.28***	0.05
LGOLGO			−0.19***	0.03	−0.21***	0.03	−0.22***	0.03
IS					0.06	0.04	0.17**	0.06
LGOIS					0.17***	0.04	0.11*	0.04
LGOLGOIS							−0.17**	0.03

*p < 0.05; **p < 0.01; ***p < 0.001.

The interaction effect between LGO and the incentive schemes of the model predicting knowledge sharing was statistically significant (β = 0.17, *p* < 0.001). This means that the threshold at which the positive relationship between LGO and knowledge sharing disappears depends on the level of incentive schemes. As shown in Step 4, the interaction effects between the quadratic term of LGO and incentive schemes were also statistically significant of the models predicting knowledge sharing (β = −0.17, *p* < 0.001), indicating that Hypotheses 3 was supported. To better understand this effect, we compared the curvilinear relationship in low-incentive schemes to that in high-incentive schemes. This was achieved by replacing the values of incentive schemes (−1.00 SD or 1.00 SD) in the model relating LGO and knowledge sharing in Step 4. That is, we used the regression coefficients to construct polynomial regression models reflecting the relationships between LGO (in standardized scores) for the low-incentive scheme and high-incentive schemes. Table 7 presents the regression coefficients for the two models and the corresponding inflection points. As can be seen, the inflection point for low-incentive scheme (1.29 SD above the mean of LGO) is much higher than that for high-incentive schemes (0.88 SD above the mean).

**TABLE 7:** Moderating effects of IS on the relationships between LGO and KS.

	Regression coefficients (B)			
LGO-KS	Intercept (B_0_)	Linear (B_1_)	Quadratic (B_2_)	Z_*inflection*_ = −B_1_/2B_2_
Low-IS (−1.00 SD)	3.80	0.18	−0.07	1.29
High-IS (1.00 SD)	4.09	0.35	−0.20	0.88

*Z_inflection_ = standardized score on the LGO scale corresponding to the inflection point of the curve reflecting the relation between LGO and KS. That is, the relation between LGO and KS starts changing direction (or reaching an asymptotic point) at this score.*

**FIGURE 2:**
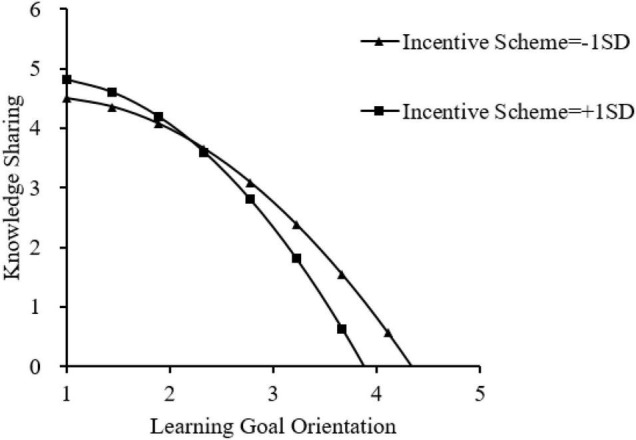
Relationships between LGO and KS.

Plots describing the combined interactive and quadratic effects of incentive schemes on KS are presented in Figure 2 (Aiken et al., 1991). The maximum point for each curve was also computed and plotted. This result provides full support for Hypothesis 3.

## Conclusion

We draw on previous studies related to LGO, knowledge sharing, and incentive schemes, together with the motivation theory and goal orientation perspective. The empirical results confirm the relationships between ICT consultants’ LGO, knowledge sharing, and incentive schemes. At a lower level, LGO could positively influence knowledge sharing; however, this influence becomes weaker as the LGO level grows and even disappears when the LGO level passes a specific threshold, i.e., LGO no longer relates to knowledge sharing among ICT consultants. This relationship is moderated by incentive schemes, whose presence could improve ICT consultants’ intention to learn and share knowledge. Moreover, we examined the interactive impact of LGO and incentive schemes on knowledge sharing among ICT consultants. Specifically, when an ICT consultant with a low level of LGO notices a low level of incentive scheme from his or her firm, he or she is motivated to share knowledge with colleagues; however, for an ICT consultant with a high level of LGO, even a high level of incentive scheme may not drive him or her to share knowledge with colleagues.

The results of this study could be interpreted as follows. First, ICT consultants who bear a LGO may offer to help and share knowledge to colleagues to demonstrate their perceived efficacy. That is, those consultants could become more confident about their problem-solving and learning abilities after learning the new knowledge. More importantly, those ICT consultants may take helping colleagues (e.g., sharing knowledge) as altruistic behavior that delivers self-enjoyment. This motivation to share knowledge is further enhanced by the reciprocal expectation that one’s knowledge sharing for the moment could receive a favor back from colleagues in the future. However, when ICT consultants’ level of LGO becomes higher, they may find it hard to share the newly acquired knowledge, which could be too abstract and complex for colleagues to understand. Knowledge sharing among ICT consultants could be further discouraged by “free-riders” who benefit from others’ knowledge and refuse to reciprocate. Eventually, the more knowledgeable ICT consultants become, the less willing they are to share specific knowledge, fearing that doing so could jeopardize their competitive advantage at work. Second, ICT consulting firms may adopt monetary and/or non-monetary incentives to change consultants’ attitudes toward knowledge sharing. Indeed, once incentive schemes satisfy ICT consultants’ physiological and psychological needs, attitudes toward knowledge sharing could become positive again. Third, incentive schemes are not the panacea for negative attitudes toward knowledge sharing. For instance, ICT consultants may evaluate the benefits developed from knowledge sharing and the costs and risks of doing so. While an ICT consultant may generously share the newly learned explicit knowledge (e.g., software usage) in exchange for rewards, he or she may fear the costs of sharing the complicated and valuable knowledge (e.g., developing algorithms to identify new opportunities or solve expensive problems). In that case, the ICT consulting firm’s existing incentive schemes may not effectively motivate the sharing of valuable knowledge.

## Contributions, Limitations, and Future Research

Knowledge sharing is critical for organizational effectiveness and innovation (Chen et al., 2018). Previous studies have investigated the impacts of employee motivation on knowledge sharing (Matzler and Mueller, 2011). As an important motivational factor, LGO could drive ICT consultants to constantly acquire new knowledge to professionally benefit themselves while exchanging with colleagues in a reciprocal manner. However, Cabrera and Cabrera (2002) remind of social dilemmas where colleagues’ free-riding behavior and the potential risks to lose competitive advantage may prevent ICT consultants from doing so. While monetary and non-monetary incentives are suggested to encourage employees to share knowledge (Brockner et al., 2006; Wolfe and Loraas, 2008; Saether, 2020), it is still unclear how ICT consultants weigh such incentives against the efforts and costs associated with sharing specific knowledge. Drawing on the motivation theory and goal orientation perspective (Dweck and Leggett, 1988; Nguyen et al., 2019), this study examines the direct and interactive impacts of LGO and incentive schemes on knowledge sharing and suggest several theoretical and managerial implications.

### Theoretical Implications

The theoretical implications of this study are twofold. First, we contribute to the motivation theory (Nguyen et al., 2019) in the context of in ICT consulting firms by examining the boundary conditions of LGO on knowledge sharing among ICT consultants. So far, studies on the impacts of LGO on knowledge sharing have assumed a linear relationship between those variables (Hassan, 2017; Nguyen et al., 2019). Our results about the curvilinear relationship between LGO and knowledge sharing provide a refined understanding of the boundary conditions through which LGO influences knowledge sharing. LGO explains the motivational processes that affect an individual’s knowledge acquisition and transfer (Dweck, 1986); it can thus explain ICT consultants’ learning and effectiveness, which are antecedents of knowledge sharing. However, too much LGO can be counterproductive; that is, as LGO reaches beyond the threshold, ICT consultants’ efforts in knowledge sharing may reduce. It is possible that excessive motivation (i.e., LGO) may turn disruptive, especially when the job-related knowledge becomes more complex or critical to employees’ competitive advantage at work. In doing so, we align with Bunderson and Sutcliffe (2003), who challenges the more-is-always-better assumption.

Second, we examined beyond the LGO by integrating incentive schemes to examine the antecedents of knowledge sharing. This may extend the goal orientation studies (Brett and VandeWalle, 1999; Bunderson and Sutcliffe, 2003) that assume motivation as a determinant to the learning effect. Ideally, monetary and non-monetary incentives could address the physiological and psychological needs (Wolfe and Loraas, 2008) of ICT consultants and shape their (knowledge) sharing behavior. Given the social dilemma situation (Cabrera and Cabrera, 2002), ICT consultants’ intention is to maximize personal gains by comparing the degree of incentives with the cost of sharing specific knowledge to colleagues (i.e., knowledge sharing dilemma). Our empirical results proved that incentive schemes motivate a learning-goal oriented ICT consultant to effectively acquire new knowledge and exchange complementary knowledge with colleagues on the condition that the incentives exceed the perceived costs of sharing knowledge. Such examinations could provide an improved understanding of the antecedents and interactive mechanisms that affect knowledge sharing.

### Managerial Implications

This study suggests some implications for managers of knowledge-intensive firms. First, as our results suggest that learning-goal orientation is an important antecedent of knowledge sharing, managers should develop incentives in their human resources management practices for their employees to develop LGO and continuously acquire and share new knowledge. However, our results suggest that managers should pay attention to the difficulty of the new knowledge. While learning-goal orientation can stimulate knowledge sharing and improve employee job performance, excessive motivation can discourage knowledge sharing, especially when the new knowledge is difficult to learn. In other words, managers could provide interventions that help employees to develop the ability to plan and monitor their learning progress. More importantly, those intervention programs should help employees develop incremental learning plans and reasonable expectations for the new knowledge to be acquired. Second, simply motivating employees to learn may not always lead to knowledge sharing, especially when the acquired knowledge can generate competitive advantages at work. We suggest that managers should develop the protection and compensation mechanisms that encourage knowledge sharing so that employees could (1) feel assured that their intellectual property will be recognized and protected and (2) determine which types of knowledge to share with colleagues and the value of doing so.

### Limitations and Future Research

This study has some limitations which suggest avenues for future research. First, the data collected through a survey method may limit the generalizability of the results regarding the relationship among LGO, knowledge sharing, and incentive schemes. Future studies could adopt alternative methods (e.g., experiments and longitudinal study) to verify the relationship among these variables and overcome the possible risks of adopting single-source data. For instance, secondary data could be adopted to evaluate employees’ job performance. Second, the Chinese and Korean participants come from a similar cultural background (i.e., collectivistic culture), which may affect the generalization of our results. For instance, future studies could examine whether the same relationship among LGO, knowledge sharing and incentive schemes could also be found in an individualistic culture. Third, this study mainly adopts the motivation theory and LGO perspective to explain the impact of motivational and incentive factors. Future studies could include other theories (e.g., social learning theory) to investigate the relationship.

## Data Availability Statement

The raw data supporting the conclusions of this article will be made available by the authors, without undue reservation.

## Ethics Statement

The studies involving human participants were reviewed and approved by the Chongqing Technology and Business University. The patients/participants provided their written informed consent to participate in this study.

## Author Contributions

All authors listed have made a substantial, direct, and intellectual contribution to the work, and approved it for publication.

## Conflict of Interest

The authors declare that the research was conducted in the absence of any commercial or financial relationships that could be construed as a potential conflict of interest.

## Publisher’s Note

All claims expressed in this article are solely those of the authors and do not necessarily represent those of their affiliated organizations, or those of the publisher, the editors and the reviewers. Any product that may be evaluated in this article, or claim that may be made by its manufacturer, is not guaranteed or endorsed by the publisher.
