# Supplementary material for: Exploring the Relationship Between Learning Goal Orientation and Knowledge-Sharing Among Information Communication Technology Consultants: The Role of Incentive Schemes
Source: Front Psychol. 2022 Feb 9;13:798668. doi: 10.3389/fpsyg.2022.798668 (PMC8863601; doi:10.3389/fpsyg.2022.798668)

**Appendix 1: Research questionnaire**

| **Knowledge sharing survey** | |
| --- | --- |
| **Thank you for taking the time to complete this survey about knowledge sharing. It aims to examine employees' motivation to learn new knowledge at work, their intention to share knowledge with colleagues, and the impacts of incentive schemes. The survey should take no more than 15 minutes to complete.**  **Your responses are confidential. To ensure anonymity, no individual or company will be identifiable in our publications.**  **Please complete all sections of the survey. You can go back at any time to revise your answers.** |  |

|  |
| --- |
| Section I. Basic information |
|  |
| **1.** Your gender * |
| ○ Male  ○ Female  ○ I prefer not to say |
|  |
| **2.** Your age* |
| ○ 25-30  ○ 31-35  ○ 36-40  ○ 41 and above |
|  |
| **3.** Your education* |
| ○ Associate degree or below  ○ Bachelor’s  ○ Master’s  ○ Doctor’s |
|  |
| **4.** Your company is based in* |
| ○ China  ○ Korea |
|  |
| **SECTION II. Please indicate your agreement with each of the following statements: '1' means you strongly disagree; '5' you strongly agree; '3' you neither agree nor disagree, and numbers in between indicate less strong views.** |
|  |
| **Your learning goal orientation** |
|  |
| **5.** Accomplishing a tough project is very satisfying.* |
| \| ○ 1 \| ○ 2 \| ○ 3 \| ○ 4 \| ○ 5 \|  \| \| --- \| --- \| --- \| --- \| --- \| --- \| |
|  |
| **6.** An important part of being a good employee is continually improving my skills. * |
| \| ○ 1 \| ○ 2 \| ○ 3 \| ○ 4 \| ○ 5 \|  \| \| --- \| --- \| --- \| --- \| --- \| --- \| |
|  |
| **7.** I put in a great deal of effort in order to learn something new related to my job.* |
| \| ○ 1 \| ○ 2 \| ○ 3 \| ○ 4 \| ○ 5 \|  \| \| --- \| --- \| --- \| --- \| --- \| --- \| |
|  |
| **Incentive schemes in your company** |
|  |
| **8.** A variable part of my pay depends on my colleagues’ assessment of the degree to which I cooperate with them.* |
| \| ○ 1 \| ○ 2 \| ○ 3 \| ○ 4 \| ○ 5 \|  \| \| --- \| --- \| --- \| --- \| --- \| --- \| |
|  |
| **9.** My bonus partly depends on the results that my team/firm achieves.* |
| \| ○ 1 \| ○ 2 \| ○ 3 \| ○ 4 \| ○ 5 \|  \| \| --- \| --- \| --- \| --- \| --- \| --- \| |
|  |
| **10.** A significant part of my salary depends on the overall performance of my colleagues.* |
| \| ○ 1 \| ○ 2 \| ○ 3 \| ○ 4 \| ○ 5 \|  \| \| --- \| --- \| --- \| --- \| --- \| --- \| |
|  |
| **11.** My company rewards and compensates those employees who help their colleagues to improve and develop.* |
| \| ○ 1 \| ○ 2 \| ○ 3 \| ○ 4 \| ○ 5 \|  \| \| --- \| --- \| --- \| --- \| --- \| --- \| |
|  |
| **Knowledge-sharing with your colleagues** |
|  |
| **12.** I often share general topics (e.g., goals & budgets) with colleagues at work.* |
| \| ○ 1 \| ○ 2 \| ○ 3 \| ○ 4 \| ○ 5 \|  \| \| --- \| --- \| --- \| --- \| --- \| --- \| |
|  |
| **13.** I often share project-specific requirements (e.g., project data, deadlines, & project rations) with colleagues at work.* |
| \| ○ 1 \| ○ 2 \| ○ 3 \| ○ 4 \| ○ 5 \|  \| \| --- \| --- \| --- \| --- \| --- \| --- \| |
|  |
| **14.** I often share methods and techniques (e.g., new techniques, methods, & failures) with colleagues at work.* |
| \| ○ 1 \| ○ 2 \| ○ 3 \| ○ 4 \| ○ 5 \|  \| \| --- \| --- \| --- \| --- \| --- \| --- \| |
|  |
| **15.** I often share important knowledge (customer insights & new opportunities) with colleagues at work.* |
| \| ○ 1 \| ○ 2 \| ○ 3 \| ○ 4 \| ○ 5 \|  \| \| --- \| --- \| --- \| --- \| --- \| --- \| |
|  |
| **16.** I often share project results (e.g., preliminary results, unexpected outcomes, & recommendations) with colleagues at work.* |
| \| ○ 1 \| ○ 2 \| ○ 3 \| ○ 4 \| ○ 5 \|  \| \| --- \| --- \| --- \| --- \| --- \| --- \| |
|  |
| **17.** Are there any other comments you would like to make about your responses or the survey in general? * |
|  |
| _________________________________ |
| This is the end of the questionnaire.  Thank you for your support! |
|  |

**Appendix 2: Tables**

**Table 1: Demographics of respondents**

| Characteristics | Category | Frequency | Percentage |
| --- | --- | --- | --- |
| Gender | Male | 215 | 55.3% |
|  | Female | 174 | 44.7% |
| Age | 25-30 | 156 | 40.1% |
|  | 31-35 | 104 | 26.7% |
|  | 36-40 | 68 | 17.5% |
|  | 41 above | 61 | 15.7% |
| Edu | Associate degree or below | 89 | 22.9% |
|  | Bachelor degree | 197 | 50.6% |
|  | Master degree | 88 | 22.6% |
|  | Doctorate degree | 15 | 3.9% |
| Nationality | China | 204 | 52.4% |
|  | South Korea | 185 | 47.6% |

**Table 2: Descriptive analysis**

| No | Codes | Items | N | Min. | Max. | Mean | SD |
| --- | --- | --- | --- | --- | --- | --- | --- |
| 1 | LGO1 | Accomplishing a tough project is very satisfying. | 389 | 1 | 5 | 3.53 | .978 |
| 2 | LGO2 | An important part of being a good employee is continually improving my skills. | 389 | 1 | 5 | 3.57 | 1.025 |
| 3 | LGO3 | I put in a great deal of effort sometimes in order to learn something new related to my job. | 389 | 1 | 5 | 3.61 | 1.016 |
| 4 | KS1 | I often share general topics (e.g., goals & budgets) with colleagues at work. | 389 | 1 | 5 | 3.69 | 1.223 |
| 5 | KS2 | I often share project specific requirements (e.g., project data, deadlines, &amp; project rations) with colleagues at work. | 389 | 1 | 5 | 3.65 | 1.136 |
| 6 | KS3 | I often share methods and techniques (e.g., new techniques, methods, & failures) with colleagues at work. | 389 | 1 | 5 | 3.85 | .901 |
| 7 | KS4 | I often share important knowledge (customer insights & new opportunities) with colleagues at work. | 389 | 1 | 5 | 3.68 | 1.172 |
| 8 | KS5 | I often share project results (e.g., preliminary results, unexpected outcomes, & recommendations) with colleagues at work. | 389 | 1 | 5 | 3.73 | .889 |
| 9 | IS1 | A variable part of my pay depends on my colleagues’ assessment of the degree to which I cooperate with them. | 389 | 1 | 5 | 3.77 | .986 |
| 10 | IS2 | My bonus partly depends on the results that my team/firm achieves. | 389 | 1 | 5 | 3.86 | .990 |
| 11 | IS3 | A significant part of my salary is due to the overall performance of my colleagues. | 389 | 1 | 5 | 3.72 | 1.051 |
| 12 | IS4 | My company rewards and compensates those employees who help their colleagues to improve and develop. | 389 | 1 | 5 | 3.77 | 1.091 |

Note: Total items, 12; Learning Goal Orientation (LGO), 3; Knowledge-sharing (KS), 5; Incentive Scheme (IS), 4.

**Table 3: Results of validity and reliability tests**

| Variables | Cronbach's Alpha | Std. Factor Loading | CR | AVE |
| --- | --- | --- | --- | --- |
| LGO | .91 | .87 | .91 | .77 |
|  |  | .86 |  |  |
|  |  | .90 |  |  |
| KS | .93 | .86 | .94 | .76 |
|  |  | .80 |  |  |
|  |  | .88 |  |  |
|  |  | .93 |  |  |
|  |  | .87 |  |  |
| IS | .88 | .79 | .89 | .66 |
|  |  | .80 |  |  |
|  |  | .84 |  |  |
|  |  | .82 |  |  |

Notes: LGO: learning goal orientation; KS: knowledge sharing; IS: incentive scheme

**Table 4: Results of correlation and discriminant validity analysis**

|  |  | Mean | SD | AVE | 1 | 2 | 3 | 4 | 5 | 6 | 7 |
| --- | --- | --- | --- | --- | --- | --- | --- | --- | --- | --- | --- |
| 1 | Gender | 1.45 | .50 | - | 1. |  |  |  |  |  |  |
| 2 | Age | 2.09 | 1.09 | - | -.03 | 1. |  |  |  |  |  |
| 3 | Nationality | 1.48 | .50 | - | .09 | -.05 | 1. |  |  |  |  |
| 4 | Edu | 2.07 | .78 | - | .00 | -.05 | .07 | 1. |  |  |  |
| 5 | LGO | 3.57 | .93 | .77 | .01 | -.05 | -.11* | -.03 | .88 |  |  |
| 6 | KS | 3.72 | .95 | .76 | -.02 | .00 | -.07 | -.03 | .38** | .87 |  |
| 7 | IS | 3.78 | .89 | .66 | -.03 | .00 | .02 | .04 | .12* | .12* | .81 |

Notes: *: p<0.05; **:p<0.01; ***: p<0.001

**Table 5: Results of discriminant validity**

| Comparison Model | χ^2^ | df | χ^2^/df | CFI | TLI | RMSEA | SRMR |
| --- | --- | --- | --- | --- | --- | --- | --- |
| Common Method Bias model | 68.455 | 50 | 1.369 | 0.995 | 0.993 | 0.031 | 0.026 |
| Three-factor model | 68.632 | 51 | 1.346 | 0.995 | 0.993 | 0.030 | 0.024 |
| Two-factor model | 748.081 | 53 | 14.115 | 0.795 | 0.744 | 0.184 | 0.117 |
| One-factor model | 1565.621 | 54 | 28.993 | 0.553 | 0.454 | 0.268 | 0.203 |

Note: Three-factor model (LGO, KS, IS); Two-factor model (LGO + KS, IS); One-factor model (LGO+KS+IS)

**Table 6: Examining the relationships between LGO and KS as Moderated by IS**

| Predictor | Knowledge sharing | | Knowledge sharing | | Knowledge sharing | | Knowledge sharing | |
| --- | --- | --- | --- | --- | --- | --- | --- | --- |
|  | β | SE. | β | SE. | β | SE. | β | SE. |
|  | Step 1 | | Step 2 | | Step 3 | | Step 4 | |
| Gender | -.01 | .10 | -.02 | .09 | -.01 | .09 | -.01 | .09 |
| Age | .00 | .04 | .02 | .04 | .02 | .04 | .02 | .04 |
| Nationality | -.07 | .10 | -.02 | .09 | -.01 | .09 | -.02 | .09 |
| Edu | -.03 | .06 | -.02 | .06 | -.03 | .06 | -.02 | .06 |
| LGO |  |  | .29*** | .05 | .28*** | .05 | .28*** | .05 |
| LGOLGO |  |  | -.19*** | .03 | -.21*** | .03 | -.22*** | .03 |
| IS |  |  |  |  | .06 | .04 | .17** | .06 |
| LGOIS |  |  |  |  | .17*** | .04 | .11* | .04 |
| LGOLGOIS |  |  |  |  |  |  | -.17** | .03 |

Notes: *: p<0.05; **:p<0.01; ***: p<0.001

**Table 7: Moderating Effects of IS on the Relationships Between LGO and KS**

|  | Regression coefficients(B) | | |  |
| --- | --- | --- | --- | --- |
| LGO-KS | Intercept(B_0_) | Linear(B_1_) | Quadratic(B_2_) | Z_inflection_=-B_1_/2B_2_ |
| Low-IS(-1.00 SD) | 3.80 | 0.18 | -0.07 | 1.29 |
| High-IS(1.00 SD) | 4.09 | 0.35 | -0.20 | 0.88 |

Note: Z_inflection_ = standardized score on the LGO scale corresponding to the inflection point of the curve reflecting the relation between LGO and KS. That is, the relation between LGO and KS starts changing direction (or reaching an asymptotic point) at this score.

**Appendix 3: Figures**

**Figure 1: Theoretical Framework**


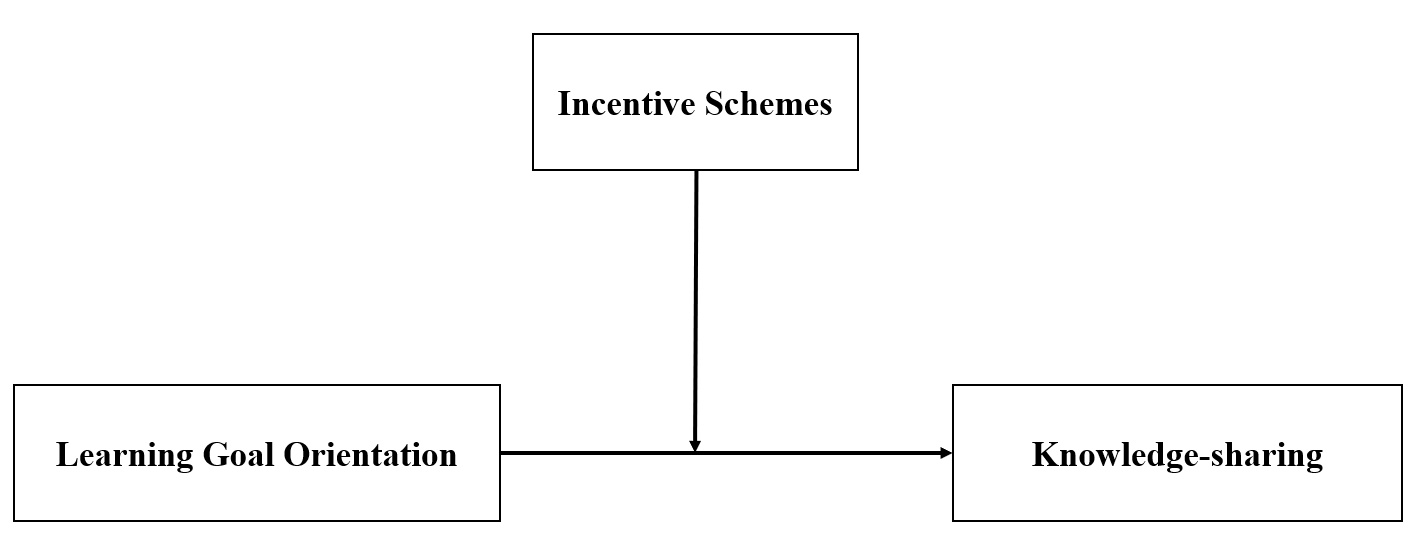


**Figure 2. Relationships between LGO and KS**


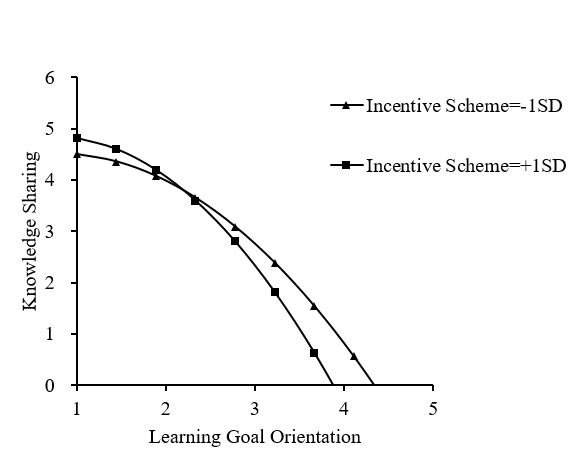

Supplement: Supplementary file 1 [file Data_Sheet_1.docx]
